# SNIPSNP: precision design of CRISPR/Cas9 knock-in reagents for variant correction and disease modeling

**DOI:** 10.1093/nar/gkag409

**Published:** 2026-06-23

**Authors:** Kornel Labun, Oline Rio, Shiva Dahal-Koirala, Anna Zofia Komisarczuk, Eivind Valen, Emma Haapaniemi

**Affiliations:** Institute of Genetics and Biotechnology, Faculty of Biology, University of Warsaw, 02-106 Warsaw, Poland; Computational Biology Unit, Department of Informatics, University of Bergen, Bergen 5006, Norway; Norwegian Centre for Molecular Biosciences and Medicine Norway, University of Oslo, Oslo 0349, Norway; Norwegian Centre for Molecular Biosciences and Medicine Norway, University of Oslo, Oslo 0349, Norway; Norwegian Centre for Molecular Biosciences and Medicine Norway, University of Oslo, Oslo 0349, Norway; Norwegian Centre for Molecular Biosciences and Medicine Norway, University of Oslo, Oslo 0349, Norway; Department of Pediatric Research, Oslo University Hospital, Oslo 0372, Norway; Computational Biology Unit, Department of Informatics, University of Bergen, Bergen 5006, Norway; Department of Biosciences, University of Oslo, Oslo 0371, Norway; Michael Sars Centre, University of Bergen, Bergen 5006, Norway; Norwegian Centre for Molecular Biosciences and Medicine Norway, University of Oslo, Oslo 0349, Norway; Department of Pediatric Research, Oslo University Hospital, Oslo 0372, Norway

## Abstract

We present SNIPSNP (crisprtools.org/snipsnp), a comprehensive bioinformatics pipeline for designing experiments for CRISPR-induced homology-directed repair (HDR). The tool addresses the critical challenge of Cas9 re-cleavage by simplifying the selection of “blocking” silent variants that are effective at inhibiting RNP binding upon donor-templated editing. SNIPSNP handles complex edits, including indels, and uses multi-objective optimization to balance editing efficiency with biological safety. From user-defined wild-type and desired HDR alleles, the pipeline identifies candidate guides, annotating them with integrated efficiency scores and genome-wide off-target assessments. Uniquely, SNIPSNP evaluates guide binding against the post-edit genome to determine whether the therapeutic variant alone disrupts repeated Cas9 recognition. When necessary, it introduces synonymous blocking variants, prioritizing PAM and seed regions to minimize re-cleavage probability and editing of the wild-type (WT) allele when editing heterozygous variants. All candidate modifications undergo safety profiling and prioritization of known benign variants from dbSNP. We experimentally validated SNIPSNP and benchmarked it on pathogenic inborn error of immunity variants in primary patient T-cells. Across loci, SNIPSNP-designed templates outperform standard “correction-only” strategies, demonstrating enhanced precision editing, and reduced re-cleavage, establishing SNIPSNP as a robust platform for genome editing and disease modeling.

## Graphical Abstract

**Graphical Abstract:**
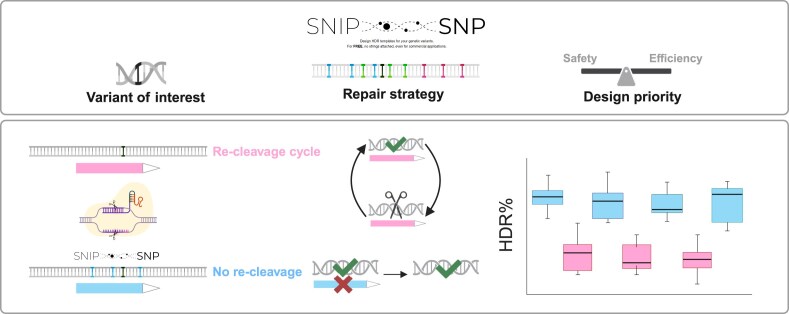

## Introduction

While the initial era of CRISPR-Cas9 genome editing was defined by gene disruption via nonhomologous end joining (NHEJ), the current translational frontier relies on high-precision correction via homology-directed repair (HDR). HDR enables the modeling and treatment of Mendelian disorders, such as inborn errors of immunity (IEI), where functional restoration often requires nucleotide-perfect knock-ins rather than stochastic indels [1, 2]. However, unlike simple gene knockout, achieving high-fidelity HDR in primary human cells presents a complex challenge of balancing cell state [3], repair pathway choice [4], local genomic context such as nucleosome breathing [5], and Cas9 ribonucleoprotein (RNP) binding kinetics [6]. The persistence of the RNP complex in the nucleus after editing leads to re-binding and re-cleavage of repaired alleles. This process favors NHEJ and gene knockout over precise HDR editing [7, 8]. In addition, mismatches introduced during HDR can be recognized by the mismatch repair (MMR) pathway, which may counteract precise editing by reversing or preventing stable donor incorporation, thereby reducing editing efficiency.

Despite the shift towards precise correction, the bioinformatics ecosystem remains optimized for gene knock-outs, relying on models trained to maximize indel frequency rather than precise integration [9, 10]. Consequently, standard design platforms neglect the thermodynamics of the post-repair state: if a donor template corrects a pathogenic variant without sufficiently disrupting the protospacer or protospacer adjacent motif (PAM), the locus remains susceptible to re-cleavage by the persistent Cas9 RNP [1, 7]. While introducing synonymous ‘blocking’ variants can abrogate re-binding, current selection strategies are largely manual, often failing to account for the complex mismatch tolerance of Cas9 in the seed region [8] or the potential for conservative transitions to generate noncanonical PAMs [11].

To mitigate re-cleavage, donor templates are frequently engineered with synonymous “blocking” variants that disrupt the PAM or seed region [8], preventing re-cutting. Such sequence differences may also be recognized by the MMR pathway during HDR. However, synonymous variants can affect the kinetics of protein translation [12] or disrupt splicing regulation by ablating exonic splicing enhancers (ESEs) and activating cryptic splice sites. This effectively converts a therapeutic repair into a novel loss-of-function allele [13, 14]. Consequently, the design of therapeutic reagents presents a multi-objective optimization problem: one must simultaneously maximize thermodynamic blocking efficiency, while minimizing potential MMR recognition, while rigorously preserving transcript integrity—a task that exceeds the capacity of manual design and requires high-dimensional modeling of coding and noncoding regulatory constraints.

Here we present SNIPSNP (crisprtools.org/snipsnp), a comprehensive bioinformatics pipeline for designing CRISPR-induced HDR reagents. The tool facilitates the selection of “blocking” synonymous single-nucleotide variants (SNVs) that are effective at inhibiting RNP binding while minimizing potential MMR recognition of donor–genome mismatches. SNIPSNP employs a dynamic coordinate-mapping engine to handle complex edits (including indels) and utilizes multi-objective optimization to balance editing efficiency against biological safety. The web interface provides intuitive visualization of the design process, featuring interactive genomic tracks where users can inspect guides, variants, and template relationships in real time. By filtering for splice-site integrity and prioritizing known benign variants, the pipeline generates templates that maximize HDR rates while preserving the transcript integrity of the target gene. The tool supports both therapeutic variant correction and disease modeling workflows.

## Materials and methods

### The SNIPSNP algorithm

SNIPSNP accepts input via a dynamic web interface or a RESTful API, requiring three primary parameters: a unique job identifier, the target species (supporting major model organisms: *Caenorhabditis elegans, Danio rerio, Drosophila melanogaster, Homo sapiens, Macaca mulatta, Mus musculus, Rattus norvegicus, Salmo salar, Sus scorfa*), and the chromosomal coordinates of the edit. Upon submission, an asynchronous validator verifies coordinate existence against the selected reference genome assembly. The core engine accepts SNVs, insertions, and deletions. To ensure compatibility with standard variant calling formats, the system automatically enforces anchor base requirements for indels and validates that the Reference (REF) allele matches the genomic sequence at the specified coordinates. For complex editing scenarios, the tool supports clustered variants, permitting multiple edits to be addressed by a single HDR template, provided the aggregate span does not exceed a user-defined window (default: 50 bp).

The core algorithmic engine operates in two sequential phases: guide RNA prioritization and HDR template optimization. First, the system identifies all valid SpCas9 PAM sites within a user-defined window (default: 30 bp) flanking the target variant. Candidate guides are ranked using a composite score that integrates on-target efficiency metrics [9, 10] and specificity profiles based on off-target binding potential assessed with CHOPOFF [18].

To engineer “blocking” variants, the algorithm maps the guide footprint onto the template sequence and generates all possible single-nucleotide substitutions at positions permissible for editing (excluding homology arms). These candidates undergo a rigorous filtering process:

1. Hard filters: Candidates are immediately discarded if they overlap splice-site windows, coding sequence start/stop boundaries, or if they violate the pan-transcript synonymous constraint. Crucially, an exception is made if the primary desired edit introduces a frameshift or stop codon; in such cases, nonsynonymous PAM-blocking variants downstream are permitted, as the transcript is already functionally disrupted.
2. Feature engineering: Surviving candidates are annotated with a Disruption Tier (based on distance from the PAM and number of SNVs) and a Safety Tier. The Safety Tier is a composite metric derived from CADD scores (v1.6) [15], AlphaGenome splice-prediction scores through the API endpoint [16], and dbSNP status [17]. Known benign variants are prioritized (Tier 1), while candidates with high predicted pathogenicity (CADD > 15 or AlphaGenome where quantile scores of splicing models are above 0.99) are downgraded. These scores are calculated for human and mouse. For other organisms, they are imputed to baseline values, and the pipeline prioritizes physical disruption and dbSNP validation.

Users select one of three optimization schemes that dictate how the algorithm weighs thermodynamic blocking against biological risk:

1. Balanced (Default): Prioritizes candidates that offer robust PAM/Seed disruption while maintaining low safety risks.
2. Disruption-First: Maximizes the probability of abrogating Cas9 binding, accepting higher predicted biological risk if necessary to prevent re-cleavage.
3. Safety-First: strictly prioritizes variants with the lowest CADD and AlphaGenome scores, sacrificing blocking efficiency if no “safe” high-disruption candidates exist.

For each guide, the algorithm employs a greedy strategy to select nonoverlapping SNVs up to the user-defined “Maximum Variants per Template” value (default: 3). The selected SNVs must satisfy the logic of the chosen optimization scheme. Finally, generated templates are assigned a Disruption Bin based on the CRISPR-MFH [18] predicted guide-template disruption efficiency, normalized with guide-guide baseline to enable between guides comparisons. If CRISPR-MFH is disabled in options or fails to return a score, the Disruption Bin is assigned by theoretical efficacy of the block (e.g. Bin 0: PAM disrupted and ≥ 2 total variants; Bin 2: PAM intact but ≥ 3 total variants). The final output ranks templates according to the user-selected scheme, ensuring the top recommendation represents the intersection of editing efficiency and biological safety. Interactive display of the templates by default selects the top five guides (by lowest off-targets) and their templates. However, users can adjust these choices in the interactive tables by checking “Display” columns.

**Figure 1.**
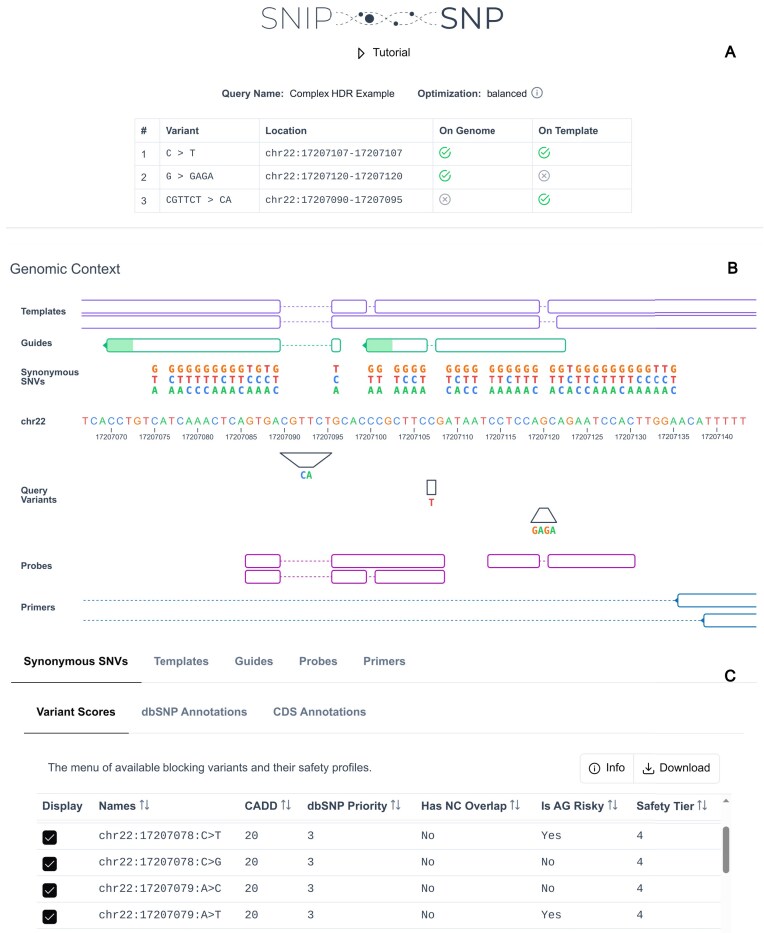
Interactive web visualization page for SNIPSNP. (**A**) Three variants (deletion, mismatch, and insertion) were used on input; they are also displayed in the “Query Variants” track in the Genomic Context view. (**B**) The Genomic Context view displays selected HDR templates, guides, blocking variants, probes, and primers as tracks, allowing for conceptual understanding of relations between reagents. Genomic Context is interactive with the tables of results below, selecting an element highlights related rows in the tables. (**C**) Tables of results come with detailed “Info” for each table and “Download” buttons. In the tables “Display” column controls which rows are displayed in the Genomic Context view.

A typical submission targeting a single locus completes in around 30 min, and users are, therefore, queued. They can close the browser and return to that url address at any point. After job completion, the frontend visualization (Fig. 1) is rendered via a dynamic D3.js-based genome browser, organizing complex genomic data into synchronized tracks that display the target sequence, evaluated variants, recommended guide/template pairs, and validation reagents. Beyond the repair template, the pipeline also designs genotyping primers and HDR-specific detection probes using Primer3-based logic, ensuring that the edited locus can be validated experimentally via polymerase chain reaction (PCR) or droplet digital PCR (ddPCR). There is also a direct integration with external genomic resources (UCSC Genome Browser, Ensembl) for independent verification of the off-targets of specific guides. All design parameters, including full oligo sequences, safety annotations, and off-target summaries, are sortable by multiple columns and downloadable in standardized formats (CSV/FASTA). Each output table contains a dedicated “Info” button with a description of each column and the software output.

## Results

### Wet-lab validation of blocking strategies

To determine the minimum sequence divergence required to prevent Cas9 re-cleavage, we performed a cell-free cleavage assay (Takara Bio sgRNA/Cas9 cleavage kit) using synthetic DNA substrates with varying degrees of complementarity to the selected single guide RNA (sgRNA). Detailed methods may be found in the supplementary materials. Consistent with previous reports describing the high mismatch tolerance of Cas9 *in vitro* [19, 20], up to three synonymous mismatches were frequently insufficient to block cleavage. Notably, a PAM transition from NGG to NAG [11] also failed to reliably prevent DNA cutting (Supplementary Fig. S1). Although such mismatches may provide partial protection *in vivo*, these data demonstrate a thermodynamic vulnerability of minimally modified donor designs, particularly under conditions of high Cas9-RNP molarity. In the guide and assay context tested here, at least four mismatches were required to fully suppress cleavage activity. All template sequences can be found in Supplementary Table S2. The nontargeting guide served as a negative control and confirmed that Cas9 does not induce cleavage in the absence of guide–target complementarity. We do, however, appreciate that a cell-free assay is highly sensitive and may overestimate the need for blocking SNVs in the absence of a cellular machinery. While also considering the potential safety aspects, we do not go above three silent SNVs per template in the software. It must also be noted that three silent SNVs in addition to the variant correction or addition will in sum be 4 base changes in the case of some sgRNAs.

**Figure 2.**
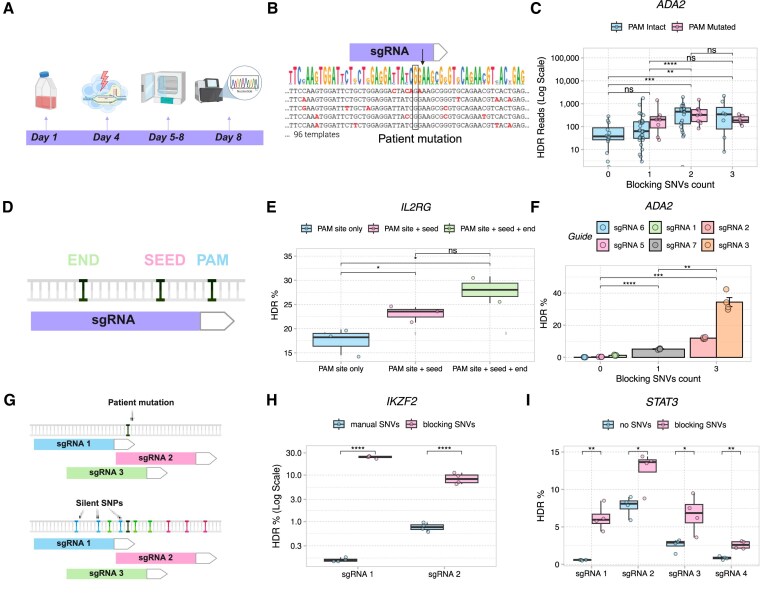
Wet-lab investigation on the effects of silent SNVs and validation of software design. (**A**) Standard protocol for nucleofection of primary T cells. PBMCs were first cultured in RPMI 1640 Medium supplemented with 10% FBS, 1% P/S, recombinant human cytokines IL-2, IL-7, IL-15, and Human CD3/CD28 T Cell Activator for three days to stimulate T cell growth. On day four, cells were nucleofected with Cas9, sgRNA, and a single-stranded DNA repair template, and kept in culture for the subsequent four days before downstream analyses after day eight. Editing efficiency was quantified by amplicon sequencing or droplet digital PCR. (**B**) Ninety-six 100bp repair templates were generated containing a unique combination of four SNVs to edit one locus (*ADA2*) with the same sgRNA. Each repair template has variations in the number of SNVs overlapping the guide sequence. The black box indicates the patient variant site, and the black arrow indicates the cut site for Cas9. (**C**) HDR reads assessed using amplicon sequencing of samples edited with the 96 unique repair templates, with blocking SNVs as the only variation between samples. (**D**) Three different placements of SNVs over the sgRNA sequence in the repair template. Over the PAM site, in the seed region (first eight bases of guide) or at the end of the guide sequence. (**E**) *IL2RG* locus edited with the same sgRNA and three different repair templates. One with a blocking SNP only in the PAM site, one with two SNPs in the PAM site and seed region, and one with SNPs in the PAM site, seed region, and end region. Editing efficiency was assessed with ddPCR. (**F**) Guide screening of the *ADA2* locus using one repair template with three SNVs across all guides. HDR was assessed with ddPCR. (**G**) Two different repair template design strategies. Introducing correction of patient variant only or correction plus three silent SNVs tailored to block each sgRNA. (**H**) Guide screening of the *IKZF2* locus using manually made SNVs versus SNVs designed by SNIPSNP software. The two design strategies were not tested in the same patient, but in two different members of the same family with the same variant. (**I**) Guide screen for *STAT3* using two different design approaches as shown in panel (G). Blocking SNVs were designed using SNIPSNP software. Statistics follow standard *t*-test where *P <.05, **P <.01, ***P <.001, ****P <.0001, ns = not significant. All data represent independent experiments with technical replicates.Created in BioRender. Rio, O. (https://BioRender.com/j9rfgev) is licensed under CC BY 4.0.

Next, we assessed how both the density and positional distribution of synonymous blocking variants influence HDR efficiency. To this end, we generated a library of 96 unique 100 bp donor templates targeting the *ADA2* p.R169Q pathogenic variant site [21] in primary human T cells from healthy donors, systematically varying the number and placement of SNVs across the protospacer and PAM-proximal region. Primary T cells were nucleofected according to protocol with Cas9 protein, an sgRNA, and a unique repair template. Amplicon sequencing revealed a positive, nonlinear correlation between the number of seed-region mismatches and precise editing efficiency (Fig. 2A–C). Donor templates incorporating “stacked” variants, defined as PAM disruption combined with additional mismatches in the seed region, significantly outperformed designs relying on PAM disruption alone.

We reproduced this effect at an independent locus, *IL2RG*, targeting the p.P58S [22] pathogenic variant site (Fig. 2D–F). T cells were edited using the same guide, Cas9 protein, and a repair template containing one, two, or three silent SNVs overlapping the guide sequence. Introduction of a second and third blocking variant within the seed region (+1 to + 8 relative to the PAM-proximal region) resulted in a marked increase in HDR efficiency compared to the single-variant control. We hypothesize this improvement may be attributed to two nonmutually exclusive mechanisms: reduced MMR activity acting on partially mismatched heteroduplex intermediates and prevention of recursive Cas9 re-cleavage (“cut-and-repair” cycling) following successful template incorporation.

Finally, we benchmarked SNIPSNP-generated donor templates against manually engineered reagents previously used in correction experiments. In patient-derived T cells harboring the *IKZF2* p.(Tyr200*) pathogenic variant [23], algorithmically optimized donor designs achieved higher overall HDR frequencies, and variant correction rates compared with the manually derived reagents [20] (Fig. 2G and H). Notably, the experiments using manual designs were performed in T cells from a different affected individual within the same family; however, both donors carried the identical pathogenic variant. Furthermore, in a therapeutic editing context targeting a *STAT3* p.K392R variant [24], SNIPSNP-designed templates enabled high-efficiency correction (Fig. 2I), demonstrating the platform’s capacity to accommodate complex, patient-specific design constraints while maintaining robust editing performance.

### Case studies on selected pathogenic variants

We analyzed the design space for the pathogenic variant ClinVar 448 033 (chr4:88 056 272 G > A). While the algorithm identified 14 theoretically possible synonymous SNVs to block the single overlapping guide, 10 of these candidates were flagged as high-risk by AlphaGenome due to high probability of splice site disruption. Additionally, four candidates carried high CADD scores, suggesting potential deleteriousness. A manual design approach, selecting a blocking variant at random, would have a high probability of compromising the transcript. In contrast, SNIPSNP successfully deprioritizes these deleterious candidates and prioritizes the remaining synonymous variants that were predicted to be splice-neutral.

The pipeline further distinguishes itself by handling complex trade-offs between theoretical safety scores and population data. In the analysis of ClinVar 362 398 (chr8:19 939 158 G > T), the design space included over 500 synonymous options. A significant portion (417 SNVs) was rejected due to splicing risks, and 501 were flagged for high CADD scores (>20). However, the engine successfully identified a specific blocking variant (rs1356370420) that disrupts the PAM sequence. Although this variant carries a CADD score of 20 with AlphaGenome prediction as dangerous for splicing, SNIPSNP prioritized it because it is a known benign polymorphism listed in dbSNP. This demonstrates the tool’s ability to override purely theoretical penalty scores when empirical population data support the safety of a specific edit.

### Safety profiling on ClinVar pathogenic variants

To further assess the risks of incorrect SNVs selection as blocking SNVs, we performed a benchmark analysis on ∼1000 randomly selected pathogenic variants retrieved from ClinVar (details in Supplementary section). Crucially, SNIPSNP ensures SNVs remain synonymous across all overlapping protein-coding transcripts. Consequently, variants that are silent in one isoform but alter the amino acid sequence in another are automatically excluded, a critical safety layer often omitted by standard tools that rely on single transcript reference models. However, in disease modeling contexts where the primary user-defined edit introduces a frameshift or premature stop codon, the algorithm intelligently relaxes this constraint for downstream sequences, as the transcript is already functionally disrupted. In addition, HDR design workflows often select synonymous variants based solely on their ability to disrupt the PAM sequence, neglecting potential biological side effects. Our analysis revealed that this approach carries significant risk. Across the entire dataset, 57% (19 973/35 320) of the theoretically possible synonymous variants were flagged as “High Risk” for splicing disruption by AlphaGenome. The model for splice sites (probability of being splice donor or acceptor) predicted 2724 loci as dangerous, splice site usage (tissue specific utilization score) had 12 094, and splice junctions (2D connection strength) had contributed the most with 17 572 marked with a quantile score above 0.99. Together, this is not surprising as ClinVar pathogenic loci are often associated with splicing disruptions [25]. Furthermore, 9% (3028/35 320) of synonymous candidates possessed CADD scores >15, indicating predicted toxicity despite no change in amino acid sequence. These findings underscore that blind selection of “silent” variants frequently results in candidates that are biologically unsafe (57%), particularly for loci associated with disease. However, cross-referencing with dbSNP, we can rescue 8% (2862/35 320) from these unsafe loci as known to be safe in the population. With slightly relaxed conditions, where we are creating a new allele at a known variable site in dbSNP, we can rescue up to 20% (6978/35 320). Every single locus we analyzed had at least one guide available for editing (NGG PAM), with a median of eleven guides per site within the default 30bp of distance from the cut site to the SNV. This demonstrates that for the vast majority of clinically relevant targets, it is possible to design HDR templates.

## Discussion

SNIPSNP addresses a gap in HDR experimental design among the current CRISPR tools. While most design platforms prioritize guide RNA selection and off-target profiling, donor template optimization remains largely manual. SNIPSNP extends guide prioritization by systematically engineering donor templates to prevent post-repair Cas9 re-cleavage, thereby stabilizing the corrected allele. Although synonymous blocking variants are known to enhance precise editing efficiency [1], their introduction can carry unintended biological risks at the target locus. SNIPSNP operationalizes this balance in systems where predictive safety models are available, by prioritizing PAM disruption and seed-proximal substitutions while minimizing unnecessary sequence divergence and enforcing transcript-level safety constraints. Unlike general guide-design tools, the platform explicitly evaluates guide binding against the post-edit genome to confirm effective disruption of Cas9 recognition.

A key limitation of the current framework is the species dependency of safety scoring. SNIPSNP integrates predictive tools such as CADD and AlphaGenome, which are well-validated for human and, to a lesser extent, mouse genomes. However, comparable models are not available for most other organisms supported by the platform. As a result, safety assessments in nonhuman systems rely on baseline or imputed values rather than organism-specific predictive modeling.

This distinction has important implications for interpretation. While safety rankings in human and mouse contexts reflect biologically informed predictions of pathogenicity and splicing disruption, scores in other species should be considered indicative and primarily reflect thermodynamic disruption and sequence constraints. Users should therefore interpret safety prioritization in nonhuman systems with caution and, where possible, complement computational design with experimental validation or organism-specific knowledge.

Advances in precision genome editing have expanded therapeutic options beyond classical CRISPR-Cas9-mediated HDR. Base editing enables specific nucleotide conversions without double-strand breaks, while prime editing supports small insertions, deletions, and all base substitutions through templated reverse transcription. These approaches reduce reliance on donor templates and lower double-strand break-associated risks, but remain constrained by PAM positioning, editing windows, complex pegRNA designs, and variable efficiency in primary human cells [26, 27]. In contrast, CRISPR-HDR remains broadly applicable across variant classes, including complex variants, and is well established in *ex vivo* cell engineering workflows such as T cell correction [2]. SNIPSNP is tailored to HDR-based correction, addressing the under-optimized problem of post-repair Cas9 re-cleavage while embedding transcript-level safety constraints into donor design. Rather than competing with base or prime editing, SNIPSNP strengthens reagent design in contexts where HDR remains the most versatile or practical strategy.

As detailed in Table 1, SNIPSNP differs from existing platforms in these main areas: (i) Synonymous PAM and seed substitutions are generated and ranked using validated mismatch tolerance landscapes, whereas most tools leave donor stabilization to manual optimization. (ii) Integration of AlphaGenome predicts splicing and regulatory impact of synonymous variants, functionality not standard in CRISPR design software. (iii) CADD scoring evaluates potential deleteriousness beyond coding preservation, adding a safety layer absent from most editing suites. (iv) Integration with dbSNP and ClinVar enables assessment of known SNVs and population-level context. The platform is freely accessible without login requirements, although an optional login via ORCID allows users to retain search history and organize projects. Together, these features position SNIPSNP as a donor-engineering platform focused on HDR stabilization rather than solely on guide efficiency.

**Table 1.** SNIPSNP uniquely integrates blocking variant design, post-edit genome evaluation, and safety profiling within a single HDR-focused workflow

Feature	SNIPSNP	HDR donor design tools (e.g. CRISPR Knock-in Designer, protoSpaceJAM)	General guide design platforms (e.g. benchling, CRISPOR)	Prime editing/base editing tools (e.g. PrimeDesign, BE-Hive)
Primary application domain	Precision HDR SNV correction; disease modeling; therapeutic editing	HDR donor construction; knock-in design; limited silent variant introduction	No – guideRNA selection for knockout, CRISPRi/a, or general targeting; HDR donor optimization is secondary or external	No – designed for BE or PE modalities, not DSB-mediated HDR engineering
guideRNA evaluation	Yes – both off-target assessment with bulges as well as efficiency prediction modeling	Partial – off-target assessment typically external; bulge tolerance not consistently evaluated	Yes – both off-target assessment with bulges as well as efficiency prediction modeling	Yes – both off-target assessment with bulges as well as efficiency prediction modeling
Cut-to-variant distance optimization	Yes – minimize cut-to-variant distance	Partial – not explicitly optimized; depends on user-selected guide	Partial (indirect) – guide ranking not explicitly optimized for HDR spatial constraints	Not applicable (BE); in PE, edit position is constrained by pegRNA design rather than HDR distance logic
Automated blocking variant design	Yes – PAM/seed-region synonymous variant generation and disruption logic	Partial – may introduce variants, but without systematic PAM/seed disruption prioritization	No – donor template optimization typically manual or user-defined	Limited – focused on pegRNA or base editor optimization, not HDR donor stabilization
Post-edit genome evaluation (Cas9 re-binding)	Yes – evaluates guide binding to edited sequence	No	No	No
Splicing impact prediction	Yes – Integrated 3 AlphaGenome (human/mouse) splicing predictions for each of the proposed SNVs	No	No	No
Variant pathogenicity/toxicity scoring	Yes – integrated CADD scoring for high risk variants (human only)	No	No	No
Population variant cross-referencing	Yes – prioritizes benign human variants, and new alleles in existing safe variants. Uses ClinVar to filter known pathogenic variants.	No	No	No
Primer design, probe design	Optimized for HDR (optional)	No or limited	No	No
Visualization	Integrated genomic view of all reagents and tables	Limited – primarily sequence-level outputs	Limited to guideRNA selection	Limited – focused on editing efficiency/outcome prediction.

Selected HDR donor-design tools (CRISPR Knock-in Designer and protoSpaceJAM) are included as closest comparators.

In contrast, existing CRISPR design tools primarily optimize guide selection or editor efficiency but do not focus on donor stabilization against re-cleavage or evaluate synonymous variant safety.

**Figure 3.**
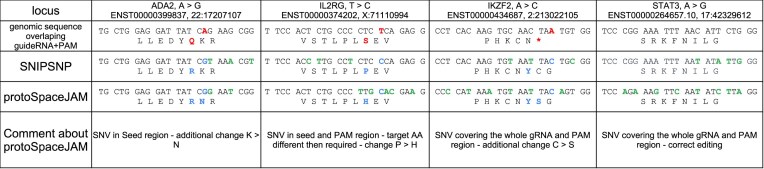
Comparison of SNIPSNP and protoSpaceJAM donor design strategies for HDR editing across selected loci. Sequences overlapping the guide RNA and PAM are shown with corresponding donor designs and amino acid translations. The Locus column indicates the desired variant, MANE transcript (Ensembl), and genomic coordinate (GRCh38) of the edited nucleotide. SNIPSNP prioritizes PAM/seed disruption while preserving coding integrity, whereas protoSpaceJAM designs may introduce unintended amino acid changes. All designs were generated using default settings for both tools. Color legend: red, target base(s); green, introduced SNVs; blue, resulting amino acid changes.

To further illustrate these differences at the sequence level, we performed a direct comparison of SNIPSNP with protoSpaceJAM [28] using representative clinically relevant loci analysed in this paper (Fig. 3). Designs were generated using default settings for both tools. CRISPR Knock-in Designer [29] was initially considered for comparison but could not be reliably executed across tested loci and was therefore excluded from this analysis.

Across all tested examples, SNIPSNP consistently generated donor templates that preserved coding integrity while introducing blocking variants in PAM and/or seed regions. In contrast, protoSpaceJAM designs frequently introduced unintended amino acid substitutions or failed to maintain synonymous constraints across the edited region. These differences highlight the importance of integrating transcript-aware safety filtering and post-edit genome evaluation into HDR donor design, particularly in therapeutic contexts where even single amino acid changes may compromise function.

The design framework is supported by experimental validation in a clinically relevant setting. HDR correction of IEI variants in both healthy donor- and patient-derived primary T cells demonstrated that donor templates incorporating synonymous blocking variants consistently enhanced precise correction efficiency compared to correction-only designs. In complementary cell-free cleavage assays, multiple mismatches were required to fully suppress Cas9 cutting, supporting our observation that combining PAM disruption with additional seed-region substitutions improves protection against re-cleavage and stabilizes HDR outcomes. The achieved correction efficiencies were therapeutically meaningful, indicating that rational donor engineering can increase precise editing without reliance on exogenous HDR enhancers. Although in the context of HDR efficiency, increasing the number of SNVs seems beneficial, we also want the software to address the safety concerns and attempt a balance between blocking gRNA re-binding and a limited number of changes. Blocking the PAM site alone should theoretically be sufficient and while our data may indicate an increased benefit in more SNVs, this requires further studies and a deeper mechanistic understanding.

Nevertheless, although SNIPSNP integrates state-of-the-art predictive tools for blocking variant prioritization and has been experimentally validated in primary T cells, broader validation across additional cell types, genomic loci, and disease contexts remains necessary. As with all computational design platforms, predicted safety and efficiency must ultimately be confirmed empirically, as editing outcomes are influenced by genomic and cellular context, as well as by experimental conditions.

## Conclusion and outlook

SNIPSNP converts a complex donor design process into a simplified, yet comprehensive workflow. By integrating guide selection, post-edit genome evaluation, blocking variants optimization, and transcript-level safety screening, it prioritizes stabilization of the corrected allele and biological integrity rather than cleavage efficiency alone. Future development will extend support to additional nucleases, incorporate locus- and cell-type performance priors, and refine regulatory modeling integration. As therapeutic genome editing advances, tools embedding mechanistic insight and safety constraints directly into reagent design will be essential. SNIPSNP represents a step toward biologically informed precision editing.

## Funding

Norges Forskningsråd (331912); Kreftforeningen (190290). Funding to pay the Open Access publication charges for this article was provided by Norges Forskningsråd (331912).

## Supplementary Material

gkag409_Supplemental_Files

## Data Availability

Zenodo link to the “HDR.design.for.CRISPR” R package that performs all designs and calculations is available under https://doi.org/10.5281/zenodo.18717152. The code’s latest version is available on https://github.com/JokingHero/HDR.design.for.CRISPR. Data used for figures is attached as supplementary tables. SNIPSNP is freely available at https://crisprtools.org/snipsnp and snipsnp.com.
