# Supplementary material for: SNIPSNP: precision design of CRISPR/Cas9 knock-in reagents for variant correction and disease modeling
Source: Nucleic Acids Res. 2026 Jun 23;54(W1):W145–53. doi: 10.1093/nar/gkag409 (PMC13355073; doi:10.1093/nar/gkag409)
Supplement: gkag409_Supplemental_Files [file gkag409_supplemental_files.zip › Supplementary Material.pdf]

Supplementary Material

ClinVar Benchmark Variant Selection

To evaluate the pipeline's performance on disease-relevant variants, we established a benchmark using the ClinVar database (GRCh38). Variants were filtered to include only single nucleotide polymorphisms (SNPs; REF and ALT length = 1 bp) annotated as either "Pathogenic" or "Likely_pathogenic". Variants with conflicting "Benign" annotations were excluded. From the resulting pool of pathogenic SNPs, exactly 1,000 variants were randomly sampled using a fixed computational seed to ensure reproducibility. Loci were not artificially restricted to coding regions or pre-filtered for CRISPR targetability; rather, the randomly selected variants were fed directly into the pipeline with a maximum allowable cut-to-mutation distance set to 30 bp. The pipeline dynamically assigned genomic context (coding vs. non-coding) using GENCODE transcript annotations. The specific variants tested, alongside their design outcomes are provided in https://github.com/JokingHero/snipsnp_figures.

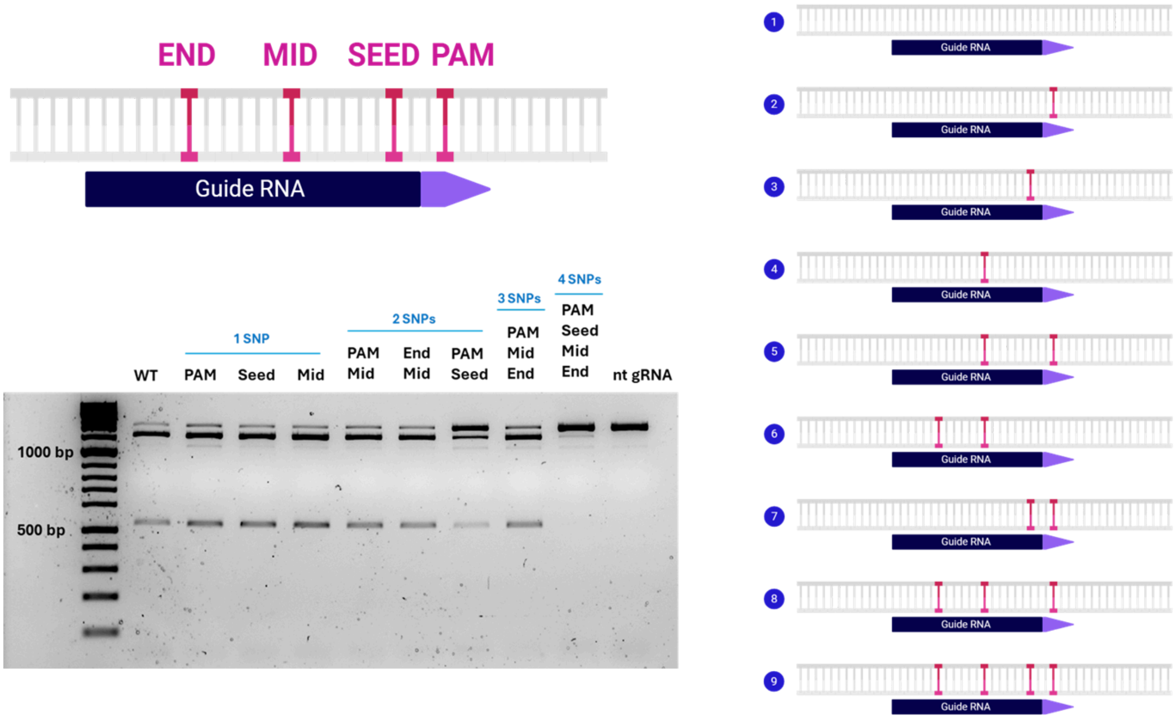

Supplementary Figure 1. Cell-free screening simulates re-cutting after adding silent SNPs. Nine different DNA templates generated with different SNP positions and combinations, then digested with annealed CAS9 and guide RNA. Samples were then run on an agarose gel (2%) to assess cutting activity. Presence of 500 bp band indicates cutting by CAS9, suggesting that only when there is a four base difference between DNA and guide RNA, will there be no cutting. All template sequences can be found in **supplementary table 2**.

Oligo sequences

All oligonucleotide sequences from **Figure 2** and **Supplementary Figure 1** can be found in **Supplementary Table 2**.

Cell culture and electroporation

PBMCs were isolated from peripheral blood of healthy donors and cryopreserved. Upon thawing the PBMCs were cultured in RPMI 1640 Medium (Thermo Fisher) supplemented with 10% FBS (Thermo Scientific) and 10% P/S or , and recombinant human cytokines IL-2 (120 U/ml), IL-7 (3 ng/ul), IL-15 (3 ng/ul) (Peprotech), and ImmunoCultTM Human CD3/CD28 T Cell Activator (15 ul/ml) (STEMCELL Technologies) for 3 nights in incubator. PBMCs from patients were cultured in

ImmunoCult (STEMCELL) with the same cocktail of cytokines. Cells were maintained at 37°C, with 21% O_2 and 5% CO_2 in a humidified incubator with an open water reservoir.

Cas9 protein, gRNA, and single-stranded oligodeoxynucleotide (ssODN) (IDT) were pre-mixed and combined with cells in the P3 primary cell Nucleofector solution (Lonza) according to kit instructions. T cells were electroporated using the Lonza 4D-Nucleofector® system with pulse codes EO-115. Each sample, consisting of one million cells in 20 ul electroporation buffer, was nucleofected with 100 pmol sgRNA, 61 pmol Cas9, and 100 pmol repair template. Cells were placed in the recovery media (RPMI with 10% FBS and 250 U/ml IL2) without P/S. Freshly prepared media (RPMI supplemented with 10% FBSm 120 U/ml IL2 (Peprotech) and 1% P/S) was added to the cell suspension 24 hrs later. Cells were cultured for four days post-electroporation before genomic DNA extraction.

Genomic DNA isolation

Genomic DNA was extracted using QIAamp DNA Blood & Tissue Kit (Qiagen). When automated processing was used, the QIAcube HT platform and QIAamp 96 DNA Kit (Qiagen) were employed, following the manufacturer’s instructions.

Droplet digital PCR and amplicon sequencing

Editing efficiency was quantified using droplet digital PCR (ddPCR) as previously described (Mamia et al). Briefly, generated droplets were subjected to amplification using a conventional thermal cycler (Bio-Rad) according to the following protocol: 1) 95 °C – 10 min, 2) 94 °C – 30 sec, 56 °C – 3 min, step repeated 42 times, 3) 98 °C – 10 min, 4) 4 °C – hold. For ddPCR, the final reaction volume was 20 μl and total input of DNA was 64 ng per sample. Data analysis was performed using QX Manager software (Bio-Rad). A minimum of 10 000 accepted droplets was used as a requirement for analysis. HDR percentage was calculated automatically using the “ratio” function in the QX manager software.

Amplicon sequencing was performed with the Illumina Miniseq version 2 platform to assess editing efficiency. Library preparation was done with a two-step PCR, the first for

locus-specific amplification and the second for adding unique indexes. Analysis of the amplicon sequencing was done using the ampliCan package.

Cell-free Cas9 cleavage assay

Cas9 cleavage activity was evaluated using a cell-free in vitro system (Takara Bio sgRNA/Cas9 cleavage kit) according to the manufacturer's instructions. Synthetic double-stranded DNA substrates (IDT) containing the respective target sequences with defined mismatches were used as cleavage templates. The dsDNA substrates (200 bp) were subcloned into the pCR™-Blunt II-TOPO® vector (Zero Blunt™ TOPO Cloning vector, Invitrogen™) and digested with BsaI (Invitrogen™) to produce two linear fragments. The larger fragment (2536 bp), containing the insert and RT template, was used in the cell-free system.

Cas9 protein was incubated with in vitro–transcribed sgRNA to allow ribonucleoprotein (RNP) complex formation prior to addition of the DNA substrate. Reactions were performed under recommended buffer and temperature conditions and terminated according to the manufacturer's protocol. Cleavage of the template resulted in two fragments of 534 bp and 2002 bp. Cleavage products were resolved by 2% agarose gel electrophoresis and visualized to assess cutting efficiency. Non-targeting sgRNA reactions were included as negative controls.

Detailed description of underlying SNIPSNP algorithm (HDR.design.for.CRISPR)

This is an algorithmic specification of how guide scoring, SNV scoring, SNV selection, and template ordering work in the HDR design pipeline. Up-to-date version of algorithmic specification is always available at the "Introduction" vignette of the HDR.design.for.CRISPR R package.

1. Inputs and Control Knobs

Primary knobs that change ranking behavior:
- `optimization_scheme` in `{balanced, disruption_first, safety_first}`
- `maximum_variants_per_template` (max introduced SNVs per template)
- `score_efficiency` (whether guide efficiency models are run)
- `crispr_mfh_based_scoring` (disable CRISPR-MFH scoring algorithm)

- `alphagenome_context` (optional tissue/context filter for AlphaGenome)
- optional annotations: dbSNP, CADD, ClinVar, AlphaGenome

2. High-Level Pipeline

1. Normalize input variants.
2. Discover candidate guides on a variant-centered sequence.
3. Optionally score guides with multiple efficiency models and aggregate ranks.
4. Assess guides for "auto-deactivation" by primary variants (>= 3 mismatches or PAM + 2 total).
5. Build candidate SNV positions for remaining active guides only.
6. Generate all possible SNVs at those positions and filter invalid/risky loci.
7. Compute SNV scoring features and assign scheme-specific `priority_group`.
8. For each guide and each requested SNV count `mpt`:
   - sort SNVs by scheme
   - greedily select non-overlapping SNVs
   - require exact cardinality (`selected_count == mpt`)
9. Build template sequence and compute template metrics.
10. Compute `disruption_bin` and apply final template sort based on scheme.

3. Guide Discovery and Scoring

3.1 Guide discovery

- Cas9 logic:
  - forward PAM: `NGG`
  - reverse PAM: `CCN`
- Each guide record contains:
  - `original`: 20mer protospacer
  - `with_pam`: 23mer protospacer+PAM

3.2 Optional guide efficiency scoring

If `score_efficiency = TRUE`, run multiple scoring models per guide.
Current model set:
- Doench 2014
- Moreno-Mateos 2015
- Labuhn 2018

Scoring failures are tolerated per model per guide:

- failed model call => `NA` for that model/guide
- other models still contribute

3.3 Aggregated guide rank

For each successful model:
1. rank guides by descending model score.
2. for each guide, collect available ranks (ignore `NA`).
3. compute the geometric mean of collected ranks.
4. `rank_by_scores` is the rank of geometric mean (lower is better).

If no model succeeds for any guide, `rank_by_scores = NA` for all guides.

4. Candidate SNV Generation

Candidate SNV positions are derived from guide-template alignment, then constrained:

1. Map guide footprint positions on template.
2. Exclude homology-arm positions (only active region is eligible).
3. Convert footprint position to `position_in_guide` (strand-aware).
4. Remove positions that overlap original protected template variants.

At each surviving position, generate all 3 possible single-nucleotide substitutions.

5. Candidate SNV Filtering and Annotation

Apply filters in sequence:

1. Remove positions near splice-site windows.
2. Remove positions near CDS start/stop boundaries.
3. Optionally remove ClinVar-overlapping positions.
4. Evaluate coding consequences across affected transcripts.
   - Keep SNVs only if every affected transcript is either:
     - synonymous, or
     - moot due to already-broken transcript state (i.e., if your desired primary edit introduces a frameshift or stop codon, the software allows non-synonymous PAM-blocking variants downstream, as the gene is already knocked out), or
     - non-coding for that SNV.

Optional annotations attached to surviving SNVs:
- dbSNP matching information
- noncoding overlap flag
- CADD score
- AlphaGenome splice-effect features

6. SNV Feature Engineering

6.1 Disruption tier (`disruption_tier`, lower is better)

Derived from `position_in_guide`:
- tier 1: `position_in_guide >= 22`
- tier 2: `>= 17`
- tier 3: `>= 13`
- tier 4: `>= 10`
- tier 5: otherwise

6.2 dbSNP priority (`dbSNP_priority`, lower is better)

- 1: known compatible dbSNP allele exists
- 2: overlapping dbSNP exists but allele is not compatible
- 3: no informative dbSNP evidence

6.3 CADD feature

- `cadd_imputed = CADD`
- if missing: impute to `15`

6.4 AlphaGenome feature

AlphaGenome evaluates 3 splicing predictors (`SPLICE_SITES`, `SPLICE_SITE_USAGE`, `SPLICE_JUNCTIONS`). Processing behavior:
- Takes the absolute effect magnitude (distance from median 0).
- Applies optional context filtering by `alphagenome_context` (tissue/biosample), taking the maximum magnitude if global.
- Missing values are replaced with `0`.

Risk evaluation (`is_ag_risky`):
An SNV is flagged as risky (`is_ag_risky = TRUE`) if the number of predictors

exceeding the `alphagenome_threshold` (default `0.99`) is greater than or equal to `splicing_count` (default `1`).

6.5 Safety tier (`safety_tier`, lower is better)

Default is tier 3, then apply rules:

1. Tier 5 if noncoding overlap exists.
2. Tier 4 if predicted risky:
   - `cadd_imputed > benign_cadd_threshold` OR
   - `is_ag_risky == TRUE`
3. Tier 2 if real low-risk evidence:
   - real CADD present, low CADD, not risky, no noncoding overlap.
4. Tier 1 if known benign dbSNP and no noncoding overlap.

Default thresholds:
- `benign_cadd_threshold = 15`
- `alphagenome_threshold = 0.99`

7. Scheme-Specific SNV Priority Group

`priority_group` is assigned from (`disruption_tier`, `safety_tier`) and scheme. Lower group is better.

7.1 `balanced`

- group 1: `d<=2 & s<=2`
- group 2: `d=3 & s<=2`
- group 3: `d<=2 & s=3`
- group 4: `d=3 & s=3`
- group 5: `d>=4 & s<=2`
- group 6: `d>=4 & s=3`
- group 7: `d<=2 & s=4`
- group 8: `s>=4 & d>=3`
- group 9: `s=5`

7.2 `disruption_first`

- group 1: `d<=2 & s<=4`
- group 2: `d=3 & s<=4`

- group 3: `d>=4 & s<=4`
- group 4: `s=5`

7.3 `safety_first`

- group 1..5 maps directly from `safety_tier` 1..5.

8. Per-Guide SNV Sorting and Selection

For each guide and each requested SNV count `mpt`:

8.1 Sort candidate SNVs by scheme

Ascending order unless noted. Note that booleans (`is_ag_risky`) sort `FALSE` (0, safe) before `TRUE` (1, risky).

- `balanced`:
  - `priority_group`
  - `disruption_tier`
  - `cadd_imputed`
  - `is_ag_risky`
  - `-position_in_guide` (higher/closer to PAM position first)

- `disruption_first`:
  - `priority_group`
  - `safety_tier`
  - `cadd_imputed`
  - `is_ag_risky`

- `safety_first`:
  - `priority_group`
  - `disruption_tier`
  - `is_ag_risky`
  - `-position_in_guide`

8.2 Greedy non-overlap selection

Traverse sorted SNVs and accept an SNV only if both are true:
- no genomic overlap with selected SNVs
- no codon-key collision with selected SNVs (`tx_id + codon_num`)

Stop at `N = mpt` or exhaustion.

8.3 Exact-cardinality requirement

If the selected SNVs count is not exactly `mpt`, that `(guide, mpt)` template is skipped.

9. Template Metrics

For each produced template:

- `snvs_introduced`: semicolon list of selected SNV ids
- `total_cadd`: sum of selected `cadd_imputed`
- `total_snp_quality_score`: sum of selected `dbSNP_priority`
- `is_ag_risky`: if any SNV is risky for splicing as predicted by alphagenome
- `any_overlaps_noncoding`: any selected SNV overlaps noncoding annotations
- disruption metrics from guide-vs-template alignment:
  - `pam_disrupted_count`
  - `seed_disrupted_count`
  - `total_disruption_count`
- CRISPR-MFH scoring:
  - `crispr_mfh_score`: raw cleavage probability
  - `baseline_crispr_mfh`: per-guide baseline score
  - `relative_crispr_mfh`: normalized score for cross-guide comparison
- alignment strings for inspection:
  - `aln_guide`
  - `aln_template`

Seed/PAM mismatch windows are strand-aware:
- plus strand:
  - PAM indices: 22-23 (position 21 is N - does not impact binding)
  - seed indices: 11-20
- minus strand:
  - PAM indices: 1-2
  - seed indices: 4-13

10. Disruption Bin and Final Template Ordering

10.1 disruption_bin (lower is better)

When crispr_mfh_based_scoring = TRUE (the default) and model output is available, the template disruption score is driven by machine learning predictions:
- relative_crispr_mfh < 0.1 -> bin 0 (Platinum)
- relative_crispr_mfh < 0.2 -> bin 1 (Gold)
- relative_crispr_mfh < 0.4 -> bin 2 (Silver)
- relative_crispr_mfh < 0.6 -> bin 3 (Bronze)
- relative_crispr_mfh < 0.8 -> bin 4 (Iron)
- relative_crispr_mfh >= 0.8 -> bin 5 (Dirt)

Override: Any template with zero introduced SNVs (total_disruption_count = 0) is forced to bin 5 (Dirt).

If crispr_mfh_based_scoring = FALSE or if model execution fails, a deterministic heuristic fallback is used based on mismatch count and PAM status:
- bin 0 (Platinum): pam_disrupted_count >= 1 AND total_disruption_count >= 2
- bin 1 (Gold): pam_disrupted_count >= 1 AND total_disruption_count == 1
- bin 2 (Silver): pam_disrupted_count == 0 AND total_disruption_count >= 3
- bin 3 (Bronze): pam_disrupted_count == 0 AND total_disruption_count == 2
- bin 4 (Iron): pam_disrupted_count == 0 AND total_disruption_count == 1
- bin 5 (Dirt): total_disruption_count == 0

Safety penalty is then applied directly to disruption rank
(except in disruption_first, where penalty is not applied):
- unsafe_snv_count = number of selected SNVs with safety_tier >= 4
- final disruption_bin = min(5, base_disruption_bin + unsafe_snv_count)
- n_snvs is derived from snvs_introduced token count.

10.2 Final ordering per `optimization_scheme`

- `balanced`:
  1. `any_overlaps_noncoding` (FALSE first)
  2. `disruption_bin`
  3. `total_snp_quality_score`
  4. `n_snvs`
  5. `is_ag_risky`

- `disruption_first`:
  1. `disruption_bin` (Ascending)
  2. `relative_crispr_mfh` (Ascending)
  3. `n_snvs` (Ascending)

4. -`total_disruption_count` (Descending; more cuts is better)

- `safety_first`:
  1. `any_overlaps_noncoding`
  2. `total_snp_quality_score`
  3. `disruption_bin`
  4. `n_snvs`

11. Determinism and Edge Behavior

Deterministic behavior relies on:
- normalized/sorted variants
- deterministic sort keys at SNV and template stages
- fixed random seed for any random components in the broader workflow

Important edge behavior:
- no valid SNV positions in active region => stop
- no valid candidate SNVs after filtering => stop
- no successful guide scorers => guide rank remains `NA`
- missing CADD => imputed neutral value
- missing AlphaGenome modalities => treated as zero contribution
